# Thermodynamic analysis of DNA hybridization signatures near mitochondrial DNA deletion breakpoints

**DOI:** 10.1016/j.isci.2021.102138

**Published:** 2021-02-04

**Authors:** Lakshmi Narayanan Lakshmanan, Zhuangli Yee, Barry Halliwell, Jan Gruber, Rudiyanto Gunawan

**Affiliations:** 1 Lee Kong Chian School of Medicine, Nanyang Technological University, Singapore, Singapore; 2 Institute for Chemical and Bioengineering, ETH Zurich, Zurich, Switzerland; 3 Department of Biological Sciences, Faculty of Science, National University of Singapore, Singapore, Singapore; 4 Department of Biochemistry, Yong Loo Lin School of Medicine, National University of Singapore, Singapore, Singapore; 5 Ageing Research Laboratory, Science Division, Yale-NUS College, Singapore, Singapore; 6 Department of Chemical and Biological Engineering, University at Buffalo, Buffalo, NY, USA

**Keywords:** Molecular Genetics, Bioinformatics, Sequence Analysis

## Abstract

Broad evidence in the literature supports double-strand breaks (DSBs) as initiators of mitochondrial DNA (mtDNA) deletion mutations. While DNA misalignment during DSB repair is commonly proposed as the mechanism by which DSBs cause deletion mutations, details such as the specific DNA repair errors are still lacking. Here, we used DNA hybridization thermodynamics to infer the sequence lengths of mtDNA misalignments that are associated with mtDNA deletions. We gathered and analyzed 9,921 previously reported mtDNA deletion breakpoints in human, rhesus monkey, mouse, rat, and *Caenorhabditis elegans*. Our analysis shows that a large fraction of mtDNA breakpoint positions can be explained by the thermodynamics of short ≤ 5-nt misalignments. The significance of short DNA misalignments supports an important role for erroneous non-homologous and micro-homology-dependent DSB repair in mtDNA deletion formation. The consistency of the results of our analysis across species further suggests a shared mode of mtDNA deletion mutagenesis.

## Highlights

• We analyzed DNA hybridization signatures of 9921 mtDNA deletions in 5 species
• A large fraction of mtDNA deletions exhibit short ≤5-nt misalignment signatures
• Our results point to erroneous non-homologous DNA end-joining repair in mutagenesis
• The agreement of results across species suggests similar deletion mutagenesis

## Graphical abstract

**Figure:**
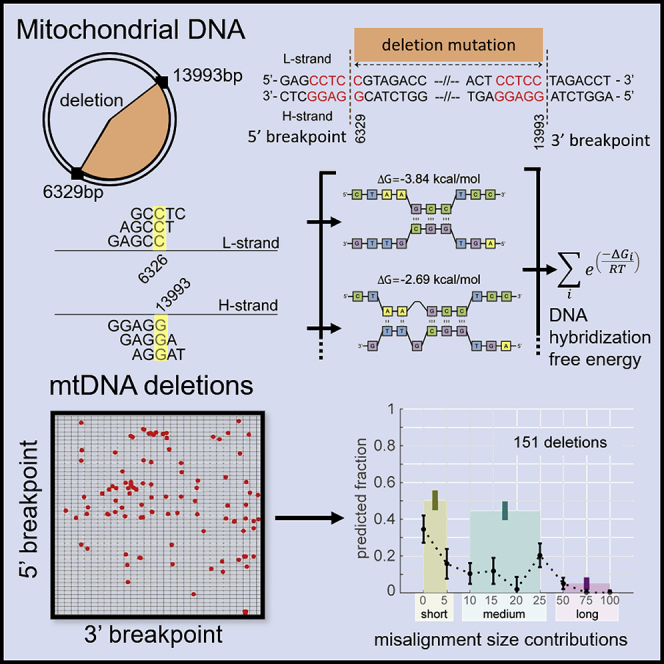

## Introduction

Pathogenic deletion mutations of mitochondrial DNA (mtDNA) typically involve the loss of several hundreds to thousands of nucleotides (nts) encoding key proteins involved in the mitochondrial electron transport chain and ATP synthesis. The accumulation of such mutant mtDNA molecules in a cell leads to mitochondrial dysfunction and ultimately cellular energy crisis. In humans, inherited mtDNA deletion mutations cause mitochondrial diseases such as Kearns-Sayre syndrome (KSS) (Holt et al., 1989; Moraes et al., 1989), chronic progressive external ophthalmoplegia (CPEO) (Holt et al., 1989; Moraes et al., 1989), and Pearson syndrome (Cormier et al., 1990; Rotig et al., 1995), and sporadic mtDNA deletions have been implicated in age-related diseases such as sarcopenia (Aiken et al., 2002). mtDNA deletions have been reported in tissues of patients suffering from Parkinson disease (Bender et al., 2006), epilepsy (Volmering et al., 2016), inclusion body myositis (Rygiel et al., 2016), Charcot-Marie-Tooth disease (Vielhaber et al., 2013), diabetes (Ballinger et al., 1992), and cancer (Zhu et al., 2004), but their role in these diseases is not clearly defined.

The mechanism of mtDNA deletion formation has been a subject of great interest and debate (Guo et al., 2010; Krishnan et al., 2008). A commonly reported feature of mtDNA deletions is the presence of direct repeat (DR) sequences precisely flanking the deletion breakpoints (Lakshmanan et al., 2012, 2015; Mita et al., 1990; Samuels et al., 2004). This observation has led to two major models of mtDNA deletion formation, namely the “slip-strand” model and the “double-strand break (DSB) repair error” model. The slip-strand model hypothesizes that mtDNA deletions occur during mtDNA replications, more specifically involving the strand-displacement mode of replication (Shoffner et al., 1989). In the strand-displacement replication, the synthesis of daughter heavy and light mtDNA strands is not synchronized. Here, the parental heavy strand is displaced and remains single stranded until roughly 65% of the daughter heavy strand is synthesized. Such a single-stranded heavy strand is prone to misalign (i.e. to hybridize with DNA sequences at an inappropriate position) with the exposed light strand, an error that involves homologous sequences such as DRs, leading to the formation of a deletion mutation (Shoffner et al., 1989). But, the slip-strand model has been questioned (Krishnan et al., 2008). One of the main objections is that this model requires the presence of “naked” single-stranded mtDNA segments during replication. However, studies on mtDNA replication have demonstrated that the displaced heavy strand is protected by mitochondrial single-strand binding proteins and often bound by RNA molecules and therefore not as open as previously thought (Holt and Reyes, 2012).

Meanwhile, the DSB repair error model, as the name suggests, attributes mtDNA deletions to erroneous repair of DSBs. Direct evidence for DSBs as an initiating step in mtDNA deletion formation comes from transgenic mouse models expressing mitochondria-targeted restriction endonuclease (Bacman et al., 2009; Fukui and Moraes, 2009; Srivastava and Moraes, 2005). Mitochondrial expression of these mtDNA targeting enzymes causes DSBs specifically at the targeted sequence locations (restriction sites) in the mtDNA. In mouse models, induction of DSBs by this method reliably promotes the formation of mtDNA deletions with breakpoints near the restriction sites. Also, mitochondrially targeted transcription activator-like effector nucleases (mito-TALEN) induced strand breaks at specific positions in human mtDNA have been shown to promote the occurrence of human mtDNA common deletion through a replisome-dependent mechanism (Phillips et al., 2016). Endogenous and exogenous DNA damaging agents that induce or promote DSBs, such as oxidative stress and ionizing radiation, have also been shown to increase the occurrence of mtDNA deletions (Ji et al., 2006; Volmering et al., 2016). Furthermore, there exists broad experimental evidence that mtDNA replication fork stalling, the collapse of which results in DSBs, promotes formation of mtDNA deletions (see, for example, Wanrooij et al. (Wanrooij and Falkenberg, 2010) and references therein). In agreement with this observation, mtDNA sequences in the neighborhood of deletion breakpoints have been shown to be enriched with sequence motifs that can promote mtDNA replication stalling, such as homopolymeric repeats (Song et al., 2003; Wanrooij et al., 2004), G-quadruplexes (Bharti et al., 2014; Dong et al., 2014), and stem loops (Damas et al., 2012).

Despite the broad evidence for an involvement of DSBs in initiating mtDNA deletions, the mechanism by which DSBs cause mtDNA deletions has yet to be established. In nuclear DNA, DSBs activate one of several DNA repair pathways, including non-homologous end joining (NHEJ), micro-homology-mediated end joining (MMEJ or alternative NHEJ), single-strand annealing (SSA), homologous recombination (HR), synthesis-dependent strand annealing, and break-induced replication (BIR) (Rodgers and McVey, 2016). *Ex vivo* experiments using mitochondrial extracts have demonstrated to different degrees of confidence the existence of mitochondrial NHEJ (Coffey and Campbell, 2000; Coffey et al., 1999; Lakshmipathy and Campbell, 1999), MMEJ (Tadi et al., 2016), and HR repair pathways (Thyagarajan et al., 1996). Mitochondrial BIR activity has been previously reported for yeast (Ling et al., 2013), but the activity of this repair pathway in mammalian mitochondria has not yet been demonstrated. One of the key features differentiating DSB repair pathways is the length of a homologous sequence (HS) around the DSB sites involved in initiating the strand annealing step of the repair process (Kent et al., 2015; Kostyrko and Mermod, 2016; McVey and Lee, 2008; Sakofsky et al., 2015). The NHEJ pathway relies on very short HSs of 0-5 nts, the MMEJ uses 0–25-nt homology (Kent et al., 2015), and the BIR pathway generally requires short (micro) HSs of at least 5-25 nts (Sakofsky et al., 2015). On the other hand, SSA and HR both utilize and require longer HSs of ≥30 nts and >100 nts, respectively (Kent et al., 2015; Kostyrko and Mermod, 2016; McVey and Lee, 2008). In addition, HR requires a template, for example, from a sister chromatid, to repair DSBs. Except for HR, errors in each of the above DSB repair pathways have been shown to cause deletion mutations in nuclear DNA (Rodgers and McVey, 2016). Therefore, in the context of the DSB repair, the length of the HSs associated with mtDNA deletions can provide both discriminating and incriminating information to deduce that a specific DSB repair pathway(s) is involved in the deletion mutagenesis (Hartlerode et al., 2016).

The DRs flanking mtDNA deletion breakpoints of an mtDNA deletion are believed to be the HS involved in the formation of that deletion. mtDNA deletions without such flanking DRs are less common and have been suggested to arise through non-homologous mechanisms (Mita et al., 1990). However, other studies have also proposed 50-100-nt sequences spanning multiple DRs around mtDNA breakpoint positions to be the alternative HS in the mtDNA deletion formation (Guo et al., 2010; Sadikovic et al., 2010). In reality, the size of mtDNA misalignments may vary among deletions, conditions, and species, i.e., there may exist an intrinsic heterogeneity in the lengths of HS involved in the deletion mutagenesis. As the aforementioned studies used only one specific sequence length (i.e. 50 or 100 nts) for studying the HS near deletion breakpoints, they were unable to provide any indication of such heterogeneity.

For this study, we developed an analytical method for identifying the distribution of mtDNA sequence lengths that are associated with a given set of mtDNA deletions. We relied on DNA hybridization thermodynamics to compute the probability of differently sized misalignments between 0 and 100 nt to occur within a range of mtDNA sequences around each mtDNA deletion breakpoint. We employed a mixture distribution model to identify the optimal distribution of mtDNA misalignment lengths that maximizes the probability of mtDNA misalignments at the observed breakpoint positions. We applied our analysis to 9,921 mtDNA deletions gathered from literature, by far the largest reported compendium, for humans, monkeys, mice, rats, and nematodes. Finally, we carried out a detailed comparative analysis of the misalignment length distributions associated with mtDNA deletions from different species and offered two scenarios by which mtDNA deletions arise from DSB repair errors.

## Results

### Characterizing the probability of DNA hybridization near breakpoints

**Figure 1:**
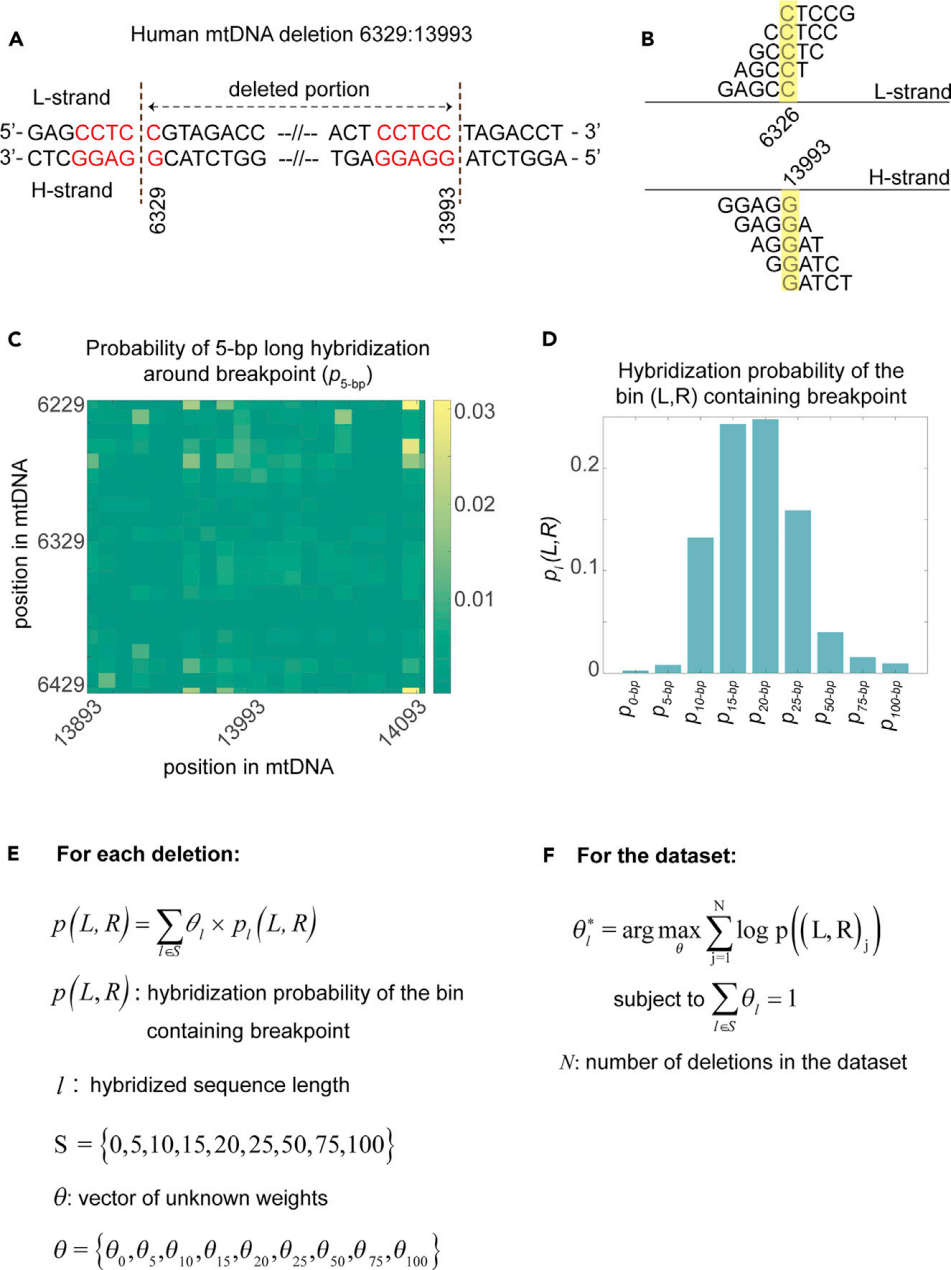
Misalignment analysis of mtDNA deletions An illustration of misalignment analysis using an example human mtDNA deletion. (A) The human mtDNA deletion has a breakpoint position pair of (6329:13,993) with a 5-nt long direct repeat flanking the breakpoint (shown in red). (B) A total of 5 × 5 = 25 distinct pairs of *l =* 5-nt sequences can form DNA duplexes that overlap the position pair (6326, 13,993) (highlighted in yellow). One of the 5-nt sequences comes from the L-strand sequence and the other from the H-strand sequence. (C) Distribution of bin propensity values for *l =* 5-nt within the 201 × 201-nt window of analysis. The color represents the relative propensity values, i.e., the propensity value of each bin divided by the sum of all bin propensity values in the window. (D) Relative bin propensity value containing the breakpoint position as a function of sequence lengths used in the analysis. (E) In the mixture model, the overall likelihood for each mtDNA deletion is computed as a weighted sum of relative bin propensity values of the mtDNA deletion breakpoint over the sequence lengths between 0 and 100 nts in the analysis. (F) For each data set, the unknown weights are calculated by maximizing the total likelihood for a given set of mtDNA deletions.

Using a human mtDNA deletion breakpoint at the nt position pair (6329:13993) as an example, we illustrate our analytical method (Figure 1). For any given mtDNA deletion, we first extract a 201-nt L-strand sequence centered on the 5′ breakpoint position and a 201-nt H-strand sequence centered on the 3′ breakpoint position. These two 201-nt sequences form the 201 nt × 201 nt, two-dimensional window of analysis centered on the mtDNA deletion breakpoint position. For the example mtDNA deletion, this window comprises L-strand sequence between 6229 and 6429 and H-strand sequence between 13,893 and 14,093, as illustrated in Figure 1. For each nt position pair in the window of analysis (i.e. one nt position from L-strand and one nt position from H-strand), we identify DNA duplexes of length *l*-nt segments—one *l-*nt segment from the L-strand and one *l-*nt segment from the H-strand—that overlap the position pair. As depicted in Figure 1B, there are *l* × *l* or 5 × 5 = 25 duplexes overlapping the nt position pair 6326-C (in L-strand) and 13993-G (in H-strand). For each of the *l* × *l* duplexes, we then compute the partition function of DNA hybridization using nearest-neighbor thermodynamics (see Methods). The partition function reflects the thermodynamic stability of duplex configurations that can form between two DNA sequences. We finally assign a propensity value for each position pair in the window of analysis. This value is equal to the sum of the partition functions over all *l* × *l* duplexes. The propensity value of a position pair is therefore indicative of the probability of the corresponding nt pair to take part in an *l*-nt long misalignment.

In the next step, we divide the window of analysis into 10 × 10-nt bins. The binning strategy is implemented to address two potential issues: (1) imprecision in the determination of the breakpoint position (e.g. due to presence of DRs) and (2) the deletion breakpoint occurring adjacent to—not within—the DNA misalignment. The misalignment propensity value for each bin is set to the sum of the propensity values of all nt position pairs in the bin. The heatmap in Figure 1C illustrates the distribution of the bin propensity values in the window of analysis for 5-nt misalignment length for the example human mtDNA deletion. Such variation is a consequence of the differences in the thermal stability of *l*-nt misalignments within the window of analysis (*l* = 5 in Figure 1C). If a given mtDNA deletion arises from a DNA misalignment of length *l*-nt, then we expect the bin containing the deletion breakpoint to be more thermally stable and thus to have a higher propensity value relative to the other bins in the window of analysis. Note that the hybridization partition function and thus the propensity value depend on the sequence length *l*.

Figure 1D depicts the relative bin propensity for the mtDNA deletion breakpoint position, i.e., the bin propensity value containing the deletion breakpoint divided by the sum of all bin propensity values in the window of analysis, as a function of the misalignment sequence length *l*. For the example mtDNA deletion, the relative bin propensity value peaks at 20 nts. While the example mtDNA deletion has a 5-nt flanking DR (see Figure 1A), our analysis suggest that this deletion could occur due to the favorable energetics of a longer misalignment of 20 nts, which includes the 5-nt DR but is not limited to it.

In this study, we tested nine sequence lengths ranging from 0 nt to 100 nts, specifically 0, 5, 10, 15, 20, 25, 50, 75, and 100 nts. These values were chosen to cover the range of misalignment lengths commonly believed to be involved in mtDNA deletions. The 0-nt length accounts for a hybridization-independent formation of mtDNA deletions, for which the (bin) propensity values are set to the same value (i.e. the uniform distribution). For higher computational efficiency, we pre-computed the partition functions of DNA hybridization for each of the sequence lengths mentioned above and for mtDNA sequences containing the majority of the reported mtDNA deletions (e.g., the entire major arc sequence of human, rhesus monkey, mouse, and rat mtDNA).

We posit that the length of DNA misalignment associated with an mtDNA deletion corresponds to the sequence length that maximizes the misalignment propensity at the mtDNA breakpoint position relative to those at the surrounding positions in the window of analysis. We employ a mixture distribution model for evaluating the relative bin propensity of a given mtDNA deletion (see Figure 1E). In this model, the relative propensity is computed as a weighted sum of the relative propensities for different misalignment lengths used in the analysis. The weights, denoted by $\theta_{l}$ (*l =* 0, 5, 10, 15, 20, 25, 50, 75, and 100 nts), are the unknown model parameters of interest. Here, we formulate a maximum likelihood estimation to obtain the unknown weights of the mixture distribution model for a group of mtDNA deletions (for example, mtDNA deletions in human aging) (see Figure 1F). The likelihood of an mtDNA deletion to occur is set to the logarithm of the relative propensity from the mixture distribution model, given the weights $\theta_{l}$. We used the interior point algorithm to obtain the optimal weights that maximize the total likelihood (MATLAB, version 2015a; MathWorks, Inc.). For a given group of mtDNA deletions, the optimal weight $\theta_{l}^{*}$ represents the proportion of mtDNA deletions in the group that are associated with the specific length *l*. We tested and validated our analysis using *in*-*silico*-generated mtDNA deletion data sets with known $\theta_{l}^{*}$ by random sampling (see Methods). Our analytical method accurately recovered the fractions of mtDNA deletions that were used to generate the *in silico* breakpoints (see Figure S1; Supplemental information). Importantly, we were able to confirm that our findings are not sensitive to the analysis window length and bin size values used for hybridization probability calculations (see Figure S2; Supplemental information).

For our analysis, we compiled an exhaustive compendium of 9,921 mtDNA deletion breakpoint positions previously reported in human, rhesus monkey, mouse, rat, and *C. elegans* and categorized these deletions into 16 groups based on the species and the experimental conditions (patient groups used, mutant strains, treatment conditions; for more details, see Table 1, Table S1; Supplemental information, and Data S1). This compendium comprised, to the best of our knowledge, the biggest data set publicly available at the time of our study.

**Table 1:** Breakpoint data sets

Data set count	Data set name	Total deletions	Source/description
1	Human MTL epilepsy	208	Mesial temporal lobe epilepsy
2	Human aging	151	Aging-associated deletions
3	Single deletion myopathy	138	Non-autosomal KSS/CPEO
4	Human POLG	85	Autosomal CPEO with POLG gene mutations
5	Inclusion body myositis	49	Inflammation in muscle
6	Pearson syndrome	34	Rare childhood disorder
7	Charcot-Marie-Tooth disease	31	Inherited nervous disorder
8	Rat aging	121	Aging-associated deletions
9	Mouse aging	63	Aging-associated deletions
10	DSB mouse	57	Mice with transgenic Pst1 and Sca1 expression
11	WT mouse (NGS)	707	Wild-type control mice
12	Sod2^+/−^ mouse (NGS)	2,169	Mice heterozygous for superoxide dismutase (*Sod2*^+/−^)
13	*TWNK*^+^ (NGS)	3,455	Wild-type mice with TWINKLE overexpression
14	Sod2^+/−^; *TWNK*^+^ (NGS)	2,355	*Sod2*^+/−^ mice with TWINKLE overexpression
15	Monkey aging	32	Aging-associated deletions
16	*C. elegans* aging	266	Aging-associated deletions
	Total	9,921	

### Hybridization signatures near deletion breakpoints in rodents and nematodes

**Figure 2:**
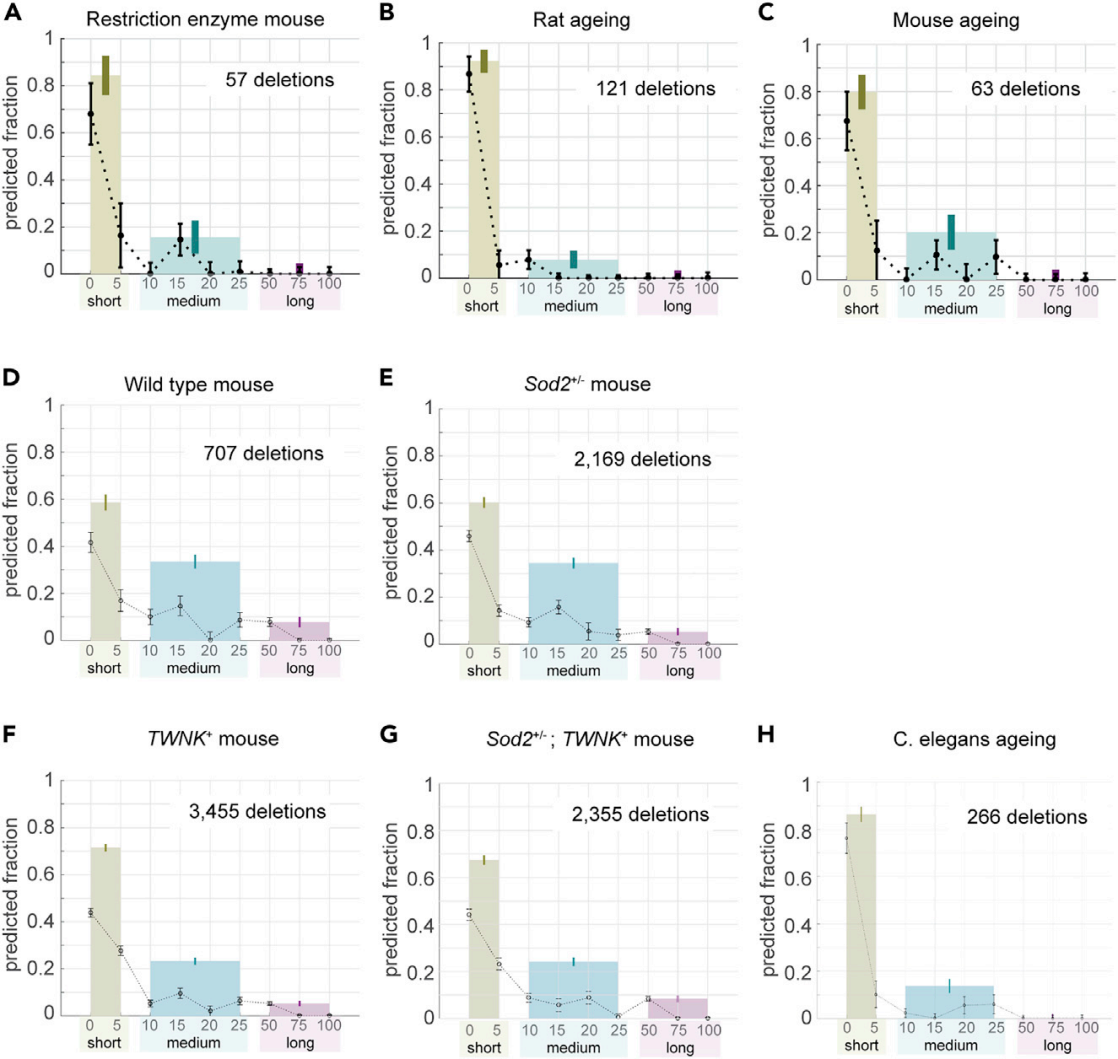
Analysis of mtDNA deletion breakpoints from rodents and nematodes Mixture model coefficients for (A) transgenic mice with mitochondrial restriction enzymes, (B) rat aging, (C) mouse aging, (D) wild-type mice (14 weeks old), (E) *Sod2*^+/−^ mice, (F) wild-type mice with TWINKLE overexpression, (G) *Sod2*^+/−^ mice with TWINKLE overexpression, and (H) *C. elegans* aging. The colored bars show the sum of the coefficients for short (0–5 nts), medium (10–25 nts), and long (≥50 nts) duplexes. The error bars indicate the sample standard deviation estimated using 100 *in silico* data sets.

The first class of mtDNA deletions in our analysis was that reported in transgenic mice expressing mitochondria-targeted restriction enzymes PstI and ScaI (see Figure 2A) (Bacman et al., 2009; Fukui and Moraes, 2009; Srivastava and Moraes, 2005). Overexpression of restriction endonucleases introduces DSBs at specific restriction sites in the mtDNA sequence and results in mtDNA deletions typically with one or both breakpoints located in the proximity to these restriction sites. Since this class of deletions derived from DSBs, the mtDNA deletion breakpoints represent the ideal case to investigate the misalignment length signature associated with DSB-induced mtDNA deletions. The outcome of our analysis as shown in Figure 2A shows a significant fraction of short misalignments (0–5 nts: 84% ± 8% [mean ± standard deviation (s.d.)]), a minor contribution from medium length (10–25 nts: 16% ± 7%), and practically no participation by longer misalignments (≥50 nts: 0% ± 4%). The significance of short misalignments is indicative of a central role of non-homologous and micro-homology-dependent repair mechanisms in the creation of this class of mtDNA deletions. On the other hand, the absence of contribution from long misalignments signifies a lack of involvement from HR repair pathway in this class of deletions.

We subsequently analyzed three different groups of mtDNA deletions found in wild-type (WT) rats and mice without any transgenic expression or diseases. The rat data set (Figure 2B) and one of the mouse data sets (Figure 2C) comprised mtDNA deletion breakpoints gathered from different reports in the literature. The second and larger mouse data set (Figure 2D) included mtDNA deletion breakpoints from a single next-generation sequencing (NGS)-based study (Pohjoismaki et al., 2013). The results of the analysis of these data sets suggest misalignment length compositions that closely resemble those of the DSB-induced mtDNA deletion data set above. Again, the largest fraction of deletions is associated with short misalignments (rat aging: 92% ± 5%, mouse aging: 80% ± 7%, WT mouse: 59% ± 3%), with the medium lengths having a smaller contribution (rat aging: 8% ± 4%, mouse aging: 20% ± 7%, WT mouse: 34% ± 3%) and the long misalignments contributing the least (rat aging: 0% ± 3%, mouse aging: 0% ± 4%, WT mouse: 8% ± 2%). The similarity in the misalignment length signature to DSB-induced mtDNA deletions supports an important role of DSBs in the formation of naturally occurring mtDNA deletions in WT mice and rats.

The aforementioned NGS study also generated mtDNA deletion breakpoints for mice heterozygous for *sod2* gene expression. Mitochondrial superoxide dismutase (Sod2) converts superoxide anions (O_2_^.-^) generated by electron transport chain into hydrogen peroxide. *Sod2*^−/−^ mice experience early postnatal lethality (Li et al., 1995; Loch et al., 2009). Meanwhile, heterozygous *Sod2*^+/−^ mice suffer increased mitochondrial oxidative damage, higher burden of mtDNA point mutations, more frequent mtDNA replication stalling, and elevated levels of recombined mtDNA molecules carrying deletions (Pohjoismaki et al., 2013). The breakpoint hotspots in *Sod2*^+/−^ also coincide with G-rich regions in the mtDNA H-strand, suggesting increased oxidative damage (8-oxoG) as the causative factor of higher levels of mtDNA deletions in the *Sod2*^+/−^ mice (Pohjoismaki et al., 2013). Our analysis of mtDNA deletion breakpoints in these *Sod2*^+/−^ mice (Figure 2E) suggests a misalignment length distribution that is similar to that of WT mice (Figures 2C and 2D). A comparison of the deletion breakpoint positions further shows that the mtDNA deletion hotspot regions in *Sod2*^+/−^ mice coincides with those in WT mice (see Figure S3; Supplemental information). The similarity in the misalignment length compositions and the deletion hotspots between WT and *Sod2*^+/−^ mice suggests that mtDNA deletions induced by increased oxidative damage and those in WT mice occur by similar mutagenesis mechanisms.

The same NGS study also demonstrated that the overexpression of TWINKLE (TWNK) helicase in *Sod2*^+/−^ mice (*Sod2*^+/−^;*TWNK*^+^) was able to rescue the oxidative stress phenotypes of *Sod2*^+/−^ (Pohjoismaki et al., 2013). While the frequency of mtDNA rearrangements, including deletions, in *Sod2*^+/−^;*TWNK*^+^ mice is lower than that in *Sod2*^+/−^ mice, it remains elevated in comparison to WT. Furthermore, TWINKLE overexpression in WT mice (*TWNK*^+^) leads to an increased level of mtDNA rearrangements (see Figure S6 in the original publication [Pohjoismaki et al., 2013]). In addition to its putative function as a helicase, TWINKLE has previously been shown to catalyze DNA recombination (Sen et al., 2012, 2016). The observed increased frequency of mtDNA rearrangements in *TWNK*^+^ mice may thus arise from higher DNA recombination activity due to TWINKLE overexpression. The misalignment length compositions of mtDNA deletions in *TWNK*^+^ and *Sod2*^+/−^; *TWNK*^+^ mice mirror each other (see Figures 2F and 2G), implying similar mechanisms of mutagenesis. In other words, TWINKLE overexpression appears to lower the frequency of *de novo* mutations but does not significantly alter the mechanism by which mtDNA deletions form in mice. But, in comparison to WT and *Sod2*^+/−^ mice, mtDNA deletions of *TWNK*^+^ and *Sod2*^+/−^; *TWNK*^+^ mice have higher fractions of the short length misalignments, specifically 5 nts (WT: 59% ± 3%, *Sod2*^+/−^: 60% ± 2%, *TWNK*^+^: 72% ± 1%, *Sod2*^+/−^; *TWNK*^+^: 67% ± 2%). A recent study demonstrated that the strand exchange activity of TWINKLE requires 3-6-nt sequence homology (Sen et al., 2016). Thus, our analysis is sensitive enough to detect the higher frequency of short 5-nt misalignment that is expected from the overexpression of TWINKLE in the above mice.

Besides rodents, we analyzed the age-related mtDNA deletion data sets recently reported for nematode *C. elegans* (Lakshmanan et al., 2018). Like mammals, mtDNA deletions in *C. elegans* are also frequently flanked by DR motifs (Lakshmanan et al., 2018; Melov et al., 1994). As depicted in Figure 2H, the misalignment length fractions for mtDNA deletions in nematodes (0–5 nts: 86% ± 3%, 10–25 nts: 14% ± 3%, ≥50 nts: 0% ± 2%) closely resemble the findings from the analyses of rodent data sets, indicating a conserved mechanism of mtDNA deletion mutagenesis between rodents and nematodes, two species evolutionarily separated by almost a billion years. While short-lived animal models are commonly used to understand the mitochondrial involvement in aging, significant differences exist between short-lived models and humans. For example, recently we have demonstrated that mtDNA deletion clonal expansion with age, a key process implicated in human sarcopenia, is undetectable in *C. elegans* (Lakshmanan et al., 2018). It would be interesting to compare whether the heterogeneity in misalignment sequence lengths observed near mtDNA deletions in short-lived animal models is comparable to that of human mtDNA deletions.

### Misalignment signatures near breakpoints in primates

**Figure 3:**
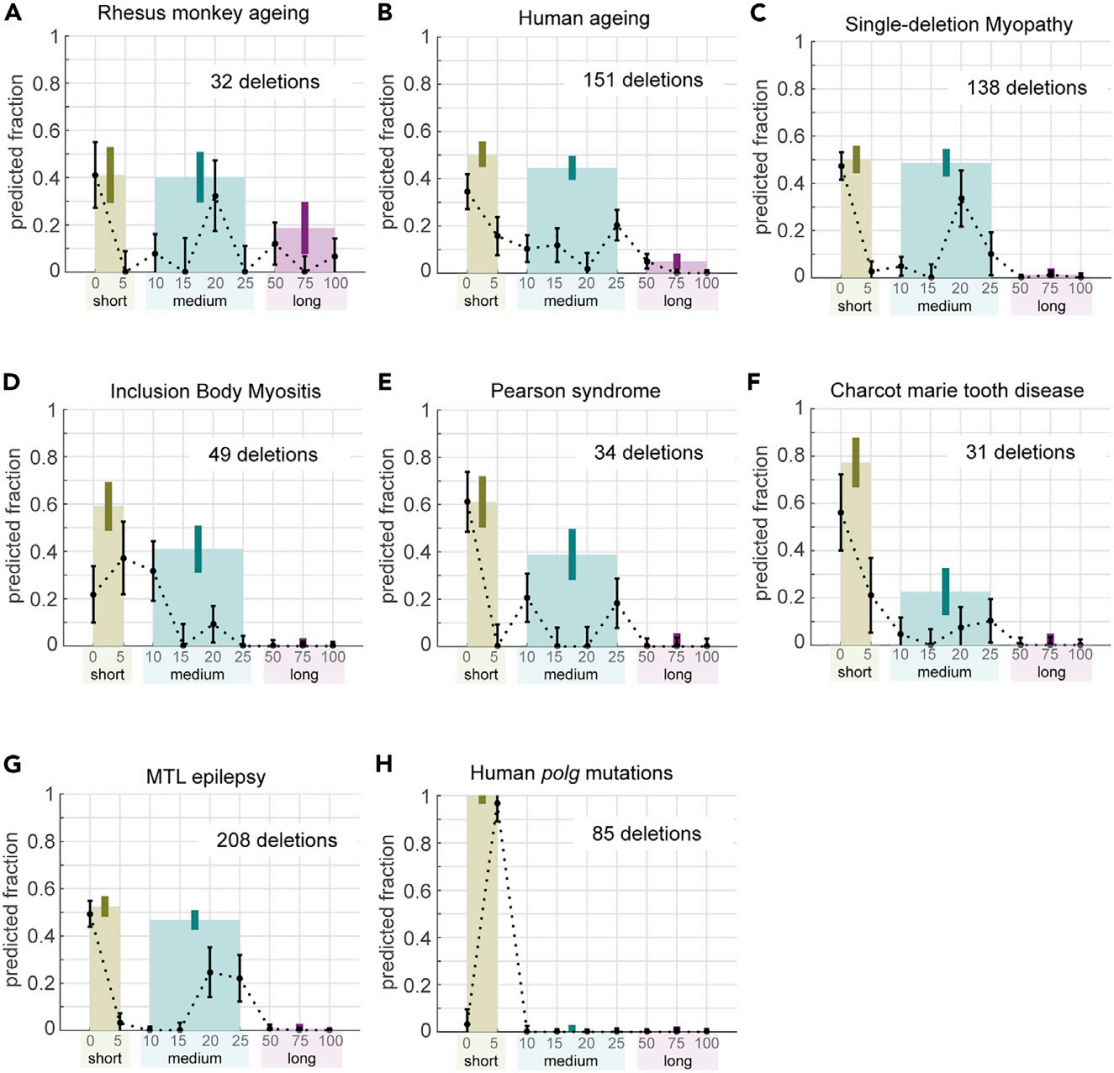
Analysis of mtDNA deletion breakpoints from primates Mixture model coefficients for (A) rhesus monkey aging, (B) human aging, (C) single deletion myopathy, (D) inclusion body myositis, (E) Pearson syndrome, (F) Charcot-Marie-Tooth disease, (G) mesial temporal lope (MTL) epilepsy, and (H) a patient with myopathy and compound *polg* gene mutations. The colored bars show the sum of the coefficients for short (0–5 nts), medium (10–25 nts), and long (≥50 nts) duplexes. The error bars indicate the sample standard deviation estimated using 100 *in silico* data sets (see Methods).

Our analysis of mtDNA deletions in aged rhesus monkey and human shows misalignment length compositions that are similar to those of rodent mtDNA deletions, albeit with nominally higher fractions of medium length misalignments (see Figures 3A and 3B; rhesus monkey: 40% ± 11%, human: 45% ± 5% vs. rat: 8% ± 4%, mouse: 20% ± 7%). In addition to mtDNA deletions associated with aging, inherited mtDNA deletions in human have also been commonly associated with autosomal myopathies and several other neuromuscular and multisystem disorders. We compiled human mtDNA deletions reported for patients with neuromuscular disorders from the literature and categorized them into 6 disease groups based on the patient description in the source articles (see Methods). Except for mtDNA deletions associated with polymerase gamma (*polg*) gene mutations, our analysis of these groups of mtDNA deletions gives compositions of misalignment lengths that are similar to those of age-related mtDNA deletions in human (see Figures 3C–3G). Thus, human mtDNA deletions during aging and in neuromuscular diseases (except autosomal *polg* mutations dataset) appear to occur by a shared mechanism.

The one exception to this pattern is the autosomal *polg* mutations (see Figure 3H). POLG is the sole DNA polymerase present in mammalian mitochondria. Mutations in *polg* cause defects in the mtDNA replication process, leading to mtDNA depletions and mutations, including deletion and point mutations (Wanrooij et al., 2004, 2007). The associated mtDNA deletions have breakpoints near homopolymeric runs (HPs) and G-quadruplexes (GQs), sequence motifs that are known to induce replication stalling (Bharti et al., 2014; Wanrooij et al., 2004). Our analysis of mtDNA deletion breakpoints from autosomal myopathy patient with compound *polg* mutations indicates that, unlike all other deletion data sets, the mtDNA deletions in these patients are associated exclusively with short HSs (see Figure 3H; 0–5 nts: 100% ± 3%). The absence of medium and long misalignments in the mutant POLG data set (10–25 nts: 0% ± 3%, ≥50 nts: 0% ± 2%) is in agreement with a previous study demonstrating a role of POLG in misalignment-dependent DSB repair in human (Phillips et al., 2016). More specifically, the study showed that the common 4,977-nt mtDNA deletion in human could be induced artificially by DSBs, through a mechanism that requires functional POLG. In addition, other DNA polymerases, such as Pol-θ, have previously been shown to take part directly in micro-homology (i.e. medium length)-mediated end-joining DSB repair in nuclear DNA (Kent et al., 2015).

In summary, our analyses of mtDNA deletion breakpoints from four mammalian species, including human, and from nematodes (*C. elegans*) point to a shared mtDNA deletion mutagenesis mechanism, involving predominantly DNA misalignments of short and medium HS lengths (0-25 nts). Meanwhile, DNA misalignments of >25 nts appear to have little or no involvement in the formation of mtDNA deletions. Except for PstI and ScaI transgenic mouse data sets, the specific event initiating the formation of mtDNA deletions in these samples remains unknown. However, the similarity of the misalignment length compositions for PstI and ScaI mice, WT mice and rats, and Sod2^+/−^ mice supports involvement of DSBs and oxidative damage in the deletion mutagenesis in rodents.

## Discussion

The formation and clonal expansion of sporadic as well as inherited mtDNA deletions ultimately causes mitochondrial respiratory dysfunction, which in human leads to a class of clinically heterogeneous disorders collectively known as mitochondrial diseases. To date, there is no effective therapeutical treatment for mitochondrial diseases due to the lack of mechanistic understanding of the disease progression. Investigations into how pathogenic mtDNA deletion mutations form and accumulate may lead to the formulation of testable hypotheses and eventually to the identification of disease-modifying targets. Over the past decades, experimental and modeling studies have provided a better understanding of the etiology of mtDNA mutation expansion (Chinnery et al., 2002; Kowald and Kirkwood, 2014; Poovathingal et al., 2009, 2012; Tam et al., 2015). In contrast, the origin and mechanism underlying mtDNA deletions are less well understood. There exists broad empirical evidence from human and model organisms that DSBs are involved in the etiology of mtDNA deletions and that deletions may result from erroneous DSB repair (Krishnan et al., 2008). However, the specific DSB repair pathway(s) involved and the mechanism by which the corresponding errors cause mtDNA deletions have yet to be established. Here, we developed a method that combines DNA hybridization thermodynamics, a mixture distribution model and maximum likelihood estimation, to determine the lengths of DNA misalignments associated with experimentally determined mtDNA deletion breakpoints in diverse species. We used the misalignment lengths as a signature for identifying the specific DSB repair mechanisms involved in mtDNA deletion formation and to examine similarities and differences in the mutagenesis mechanisms of mtDNA deletions from different species and causes.

Our analytical method overcomes some of the limitations in studying mtDNA deletion formation by only looking at DRs in the direct proximity of the reported deletion breakpoints. One limitation is associated with the fact that a large fraction of mtDNA sequences is involved in DRs (Lakshmanan et al., 2012; Samuels et al., 2004). As we have shown in a previous study, the presence of DRs around deletion breakpoints may arise simple by chance and does not unequivocally support the causal role of DRs in mtDNA deletion mutagenesis (Lakshmanan et al., 2012, 2015). In addition, the position of DRs with respect to the breakpoints may be imprecise due to technical errors during polymerase chain reaction (PCR) amplification and sequencing. For the deletion breakpoints considered in this study, many do not have precisely flanking DRs. Further, besides a few well-known deletion breakpoints, such as the 4977-bp mtDNA common deletion in human that is flanked by a 13-bp DR, the majority of the reported deletion breakpoints in the mtDNA major arcs are associated with much shorter DRs. As we reported before, a large majority of short DRs (≤5 bp) in mtDNA have positive free energies for DNA hybridization and hence in principle do not spontaneously form thermally stable duplexes (Lakshmanan et al., 2012). For these reasons, 100-bp imperfect DNA duplexes of sequences around the deletion breakpoints that may include multiple short DRs have previously been postulated to be the causative DNA misalignment involved in mtDNA deletions (Guo et al., 2010). While we also consider such imperfect duplexes, our method accounts for a range of duplex lengths, thus enabling a more precise characterization of the relevant DNA misalignments involved in mtDNA deletion formation.

Our analysis of mtDNA deletions across five species (human, rhesus monkey, rat, mouse, and *C. elegans*) suggests that the deletion breakpoint positions are most consistent with a mutagenesis mechanism that is driven by the thermodynamics of short to medium length DNA misalignments (0-25 nts). The relative contributions from longer misalignments vary slightly among species and conditions but, in general, are low. Assuming that mtDNA deletions indeed arise by errors during DSB repairs, the significance of short and medium DNA misalignments implicates NHEJ and micro-homology-dependent mechanisms (e.g. MMEJ) as key drivers of deletion formation in all species. NHEJ is generally known as the fastest and the most common route of repairing DSBs *in vivo* (Dueva and Iliakis, 2013). Our findings thus point to mitochondrial DSB repair, specifically NHEJ, as an attractive target for therapeutics for preventing or reducing the formation of mtDNA deletions.

At the same time, the small contribution from long misalignments excludes HR as an important source of mtDNA deletions. The lack of HR involvement in mtDNA deletion formation is in good agreement with two well-known aspects of the HR repair in mitochondria. First, HR is generally considered an error-free mechanism as the repair uses a template DNA molecule (Rodgers and McVey, 2016). Second, individual mtDNA molecules are typically isolated into separate nucleoids, limiting inter-molecular recombination that is necessary for HR repair (Kazak et al., 2012). While our analyses do not exonerate replication slippage as a mechanism of deletion formation, one might expect higher contributions from longer and more thermodynamically stable misalignments if deletions were to occur predominantly by the slip-strand mechanism (Bzymek and Lovett, 2001). Moreover, computational validation experiments show that our findings are not sensitive to the length of the analysis window and the length of the bins used in the analysis (Figure S2; Supplemental information). Also, applying our analysis to mtDNA deletions from individual reports separately produces the same general findings as reported above (see Figure S4; Supplemental information).

In contrast to our finding on the lack of involvement of >25-nt misalignments, a previous study by Guo *et al.* using mtDNA deletion breakpoints from the human frontal cortex found that the breakpoints are over-represented in regions of mtDNA sequences that can form highly stable 100-nt misalignments (Guo et al., 2010). While the study by Guo *et al.* and our study were based on DNA hybridization thermodynamics, our analysis differs from the one in the study by Guo *et al.* in two major aspects: (1) Our analysis accounts for all feasible duplex configurations associated with any given duplex of *l*-nt sequences, whereas Guo *et al.*’s analysis uses only the duplex configuration corresponding to the minimum free energy value. (2) Importantly, our analysis also takes into consideration DNA misalignments of different lengths, whereas Guo *et al.*’s analysis uses only pre-selected single length (100-nt) long misalignments. When we repeated the analysis of Guo *et al.* using the same breakpoints but allowing for shorter misalignment lengths, more specifically 5, 10, 25, 50, and 100 nts, we observed over-representation of deletion breakpoints in regions of mtDNA forming stable duplexes for each of these lengths, suggesting that there is, in fact, no contradiction between the two studies insofar as both methods identify the same sequences (see Figure S5; Supplemental information).

There still exists an obvious inconsistency between the 2–25-bp short deletions commonly caused by NHEJ/MMEJ errors and the 1000s of base-pair-long deletions observed in mtDNA. Despite the rarity of large deletions (>1000bp) resulting from erroneous NHEJ/MMEJ repair, previous studies of nuclear DNA have shown a scenario involving multiple DSBs by which erroneous DSB repairs could cause such deletions (Schwer et al., 2016; Xiong et al., 2015). We therefore posit that multiple DSBs (at least two) on a single mtDNA molecule could initiate the formation of a large deletion, where the DNA ends from two distinct DSBs are misjoined by NHEJ/MMEJ repair. Any two distantly located DSBs on an mtDNA molecule might come into close proximity to each other because of the folding and packing of mtDNA by mitochondrial transcription factor A (TFAM) (Gustafsson et al., 2016). Alternatively, misjoining of distant DSB ends could also occur if the mtDNA sequence in between these ends is degraded. The recombination between two mtDNA molecules, each with a DSB, may also result in a deletion mutation (Bacman et al., 2009). But such events have been shown to take place only very rarely *in vivo* (Bacman et al., 2009) and therefore are unlikely to contribute significantly to mtDNA deletion formation under physiological conditions.

An alternative scenario for lengthy mtDNA deletion mutagenesis is a single DSB invading a distant but accessible open region in mtDNA, similar to the explanation for D-loop hotspot deletions proposed in the study by Srivastava et al. (Srivastava and Moraes, 2005). The immediate question here is whether there exists a possible mitochondrial protein that is able to assist the strand invasion. Recent studies have demonstrated that TWINKLE helicase possesses several DNA modifying capacities including strand invasion (or strand exchange) (Sen et al., 2012, 2016). Consistent with the importance of short to medium HSs in our analyses, the efficiency of the strand exchange assisted by TWINKLE depends strongly on the sequence complementarity of the first 3-6 nts of the invading strand (Sen et al., 2012, 2016). Finally, there could be other, yet uncharacterized, micro-homology-dependent repair and mutagenesis mechanisms in mitochondria. For instance, a recent study reported that a strand break adjacent to the 5′ 13-nt DR flanking the common deletion in human mtDNA can induce the formation of the common deletion through a yet-to-be-characterized process that is independent of the canonical NHEJ and MMEJ enzymes (Phillips et al., 2016). Based on *in vitro* replication studies using single-stranded DNA templates with 13-nt DR flanking the common deletion in human mtDNA, another recent study has suggested that mtDNA deletions could occur during mtDNA replication using copy choice recombination process (Persson et al., 2019). Both these recent studies focused primarily on human mtDNA common deletion flanked by 13-nt DR. However, it is not immediately clear whether the remaining vast majority of mtDNA deletions with either short or no DRs could be explained by the mechanisms suggested in these recent studies (Persson et al., 2019; Phillips et al., 2016).

### Limitations of the study

The probabilistic mixture model approach proposed in this work is a fundamentally rigorous approach to identify the length of thermodynamically stable misalignments surrounding deletion breakpoints. But the current modeling framework has 2 limitations: (1) it cannot differentiate between two or more deletion mutagenesis mechanisms involving similar misalignment lengths and (2) because of the binning, this approach cannot pinpoint the orientation of misalignments relative to the breakpoints.

### Resource availability

#### Lead contact

Further information and requests for resources should be directed to and will be fulfilled by the lead contact, Dr. Rudiyanto Gunawan (rgunawan@buffalo.edu)

#### Materials availability

This study did not generate new unique reagents.

#### Data and code availability

Data sets used for this study are provided as Data S1. Codes used for generating result figures can be downloaded from the GitHub webpage: https://github.com/CABSEL/Mitochondrial-DNA-Deletion-Breakpoints-Analysis.

## Methods

All methods can be found in the accompanying Transparent methods supplemental file.

## Supplemental information

Document S1. Transparent methods, Figures S1–S5, and Table S1

Data S1. Mitochondrial DNA breakpoint positions, related to Table 1
