# Supplementary material for: Thermodynamic analysis of DNA hybridization signatures near mitochondrial DNA deletion breakpoints
Source: iScience. 2021 Feb 4;24(3):102138. doi: 10.1016/j.isci.2021.102138 (PMC7900216; doi:10.1016/j.isci.2021.102138)
Supplement: Document S1. Transparent methods, Figures S1–S5, and Table S1 [file mmc1.pdf]

iScience, Volume *24*

Supplemental Information

Thermodynamic analysis of DNA hybridization signatures near mitochondrial DNA deletion breakpoints

Lakshmi Narayanan Lakshmanan, Zhuangli Yee, Barry Halliwell, Jan Gruber, and Rudiyanto Gunawan

Supplemental Information

Figure S1. Related to Figure 1

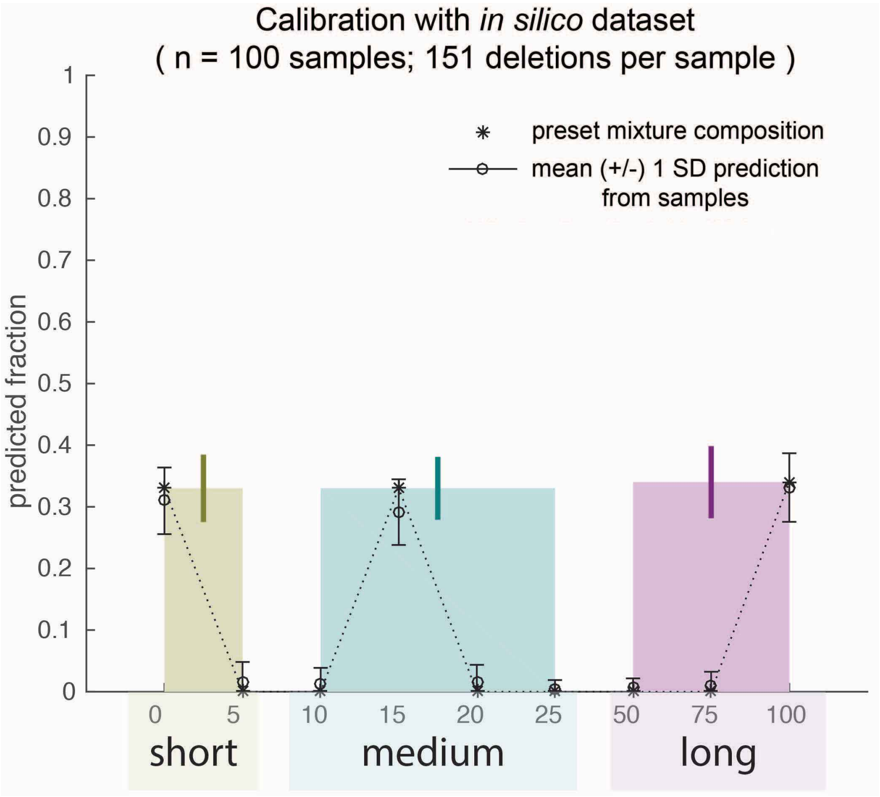

Figure S1. Mixture model analysis of *in silico* breakpoints datasets.

In silico random breakpoint datasets were generated using 3 misalignment length components with the following length composition: 33% 0-nt, 33% 15-nt and 34% 100-nt, based on human mtDNA length-specific likelihood values. Each *in silico* dataset (out of 100 datasets) comprises 151 deletions, the sample size of human aging dataset. The mixture model and the maximum likelihood estimation accurately recover the composition used to generate the *in silico* datasets. The true fractions (asterisk) and the mean ± standard deviation of the maximum likelihood estimates (circle and error bar) of the 100 *in silico* datasets are shown.

Figure S2. Related to Figure 1

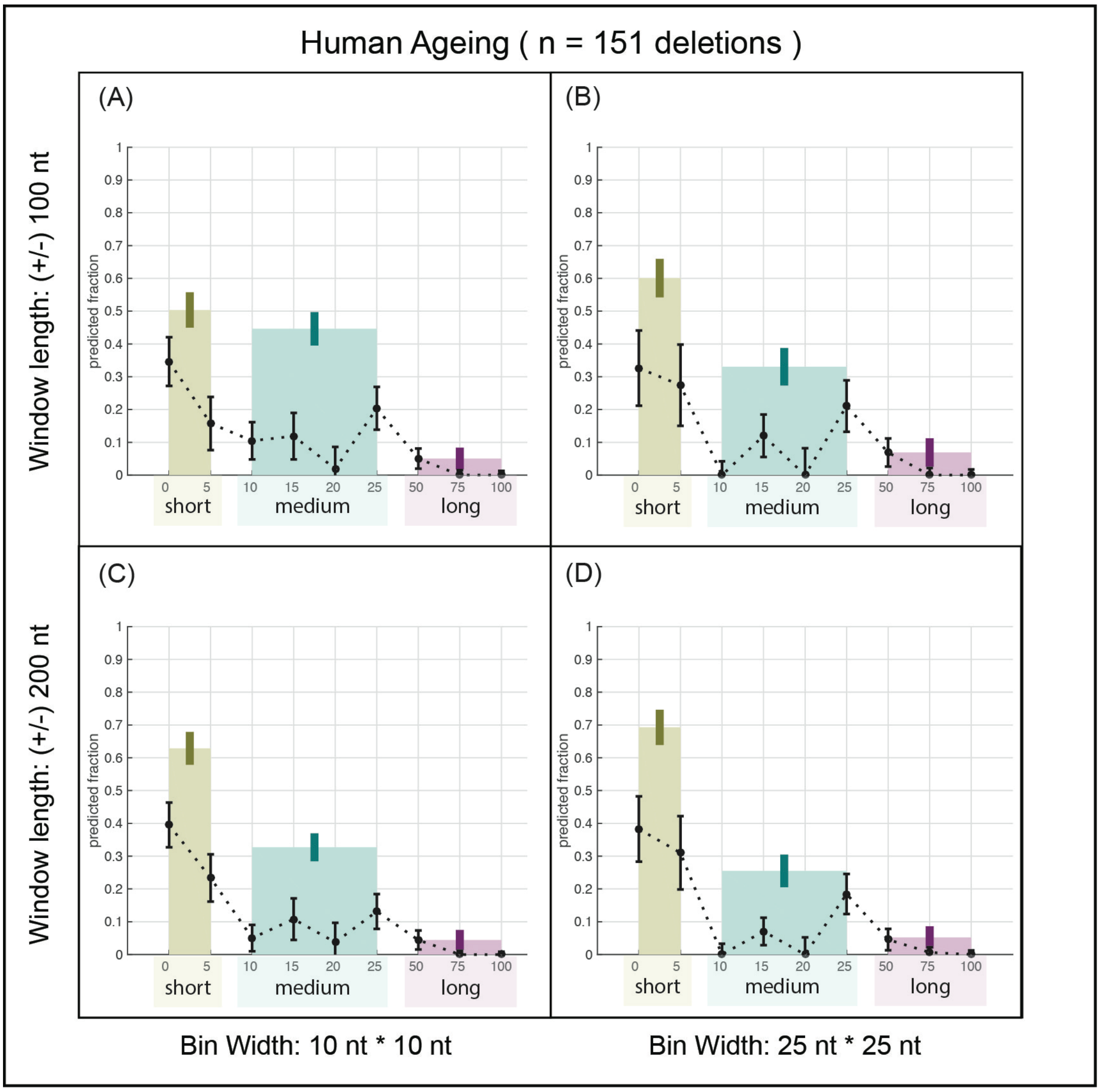

Figure S2. Mixture model analysis using differently sized window of analysis and bins.

We compared the maximum likelihood fraction θ_i^* of the mixture distribution model analysis using (A) ±100-nt window, 10-nt × 10-nt bin (default in main text), (B) ±100-nt window, 25-nt × 25-nt bin, (C) ±200-nt window, 10-nt × 10-nt bin and (D) ±200-nt window, 25-nt × 25-nt bin. The optimal fractions θ_i^* vary only slightly with the analysis window lengths and the bin sizes.

Figure S3. Related to Figure 2

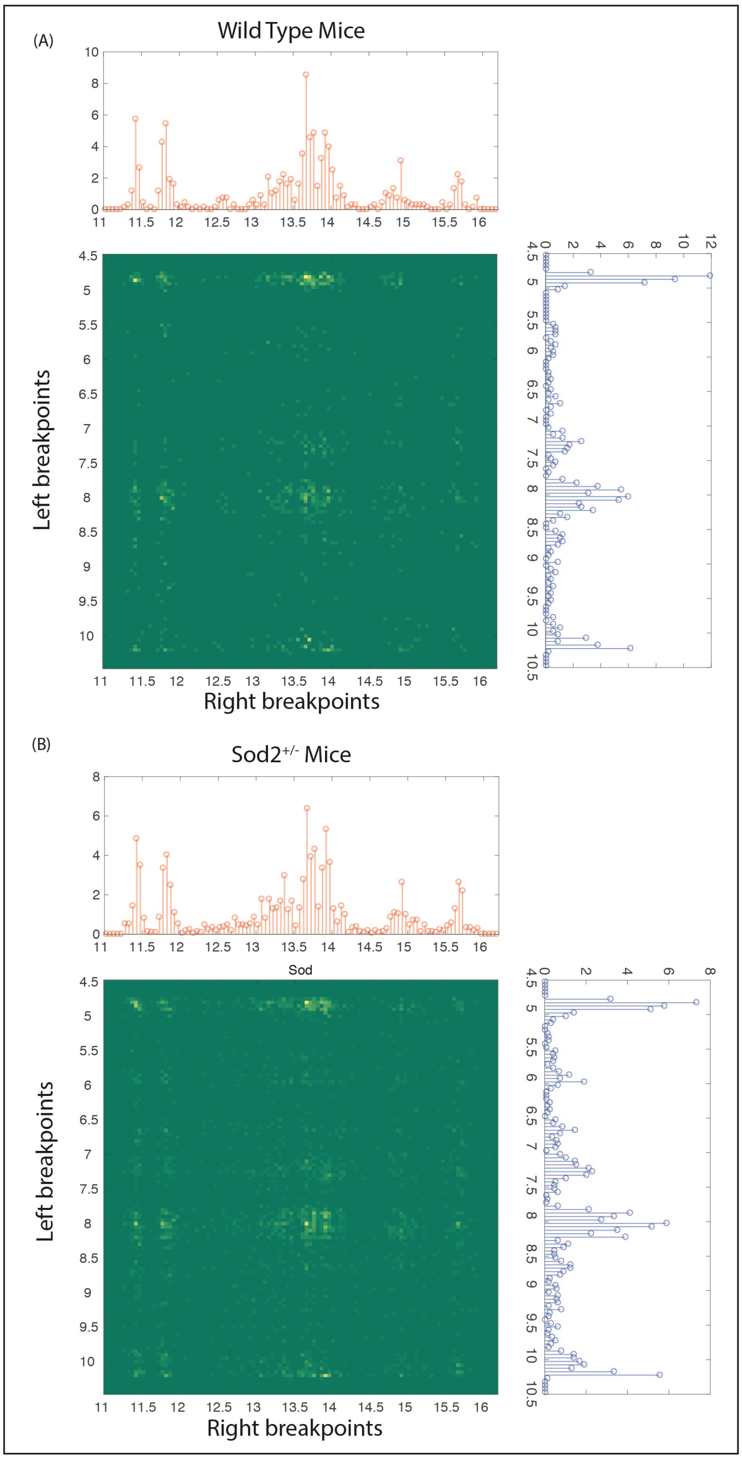

Figure S3. **Distributions of breakpoint positions**

(A) wild type mice and (B) *Sod2*$^{+/-}$ mice. The location of hotspots (i.e. peak positions of breakpoint distributions) are similar in both wild type and *Sod2*$^{+/-}$ mice, supporting the key role of oxidative damage in the formation of mtDNA deletions in wild type mice. The color intensity in the heat maps corresponds to the frequency of mtDNA deletions.

Figure S4. Related to Figures 2 and 3

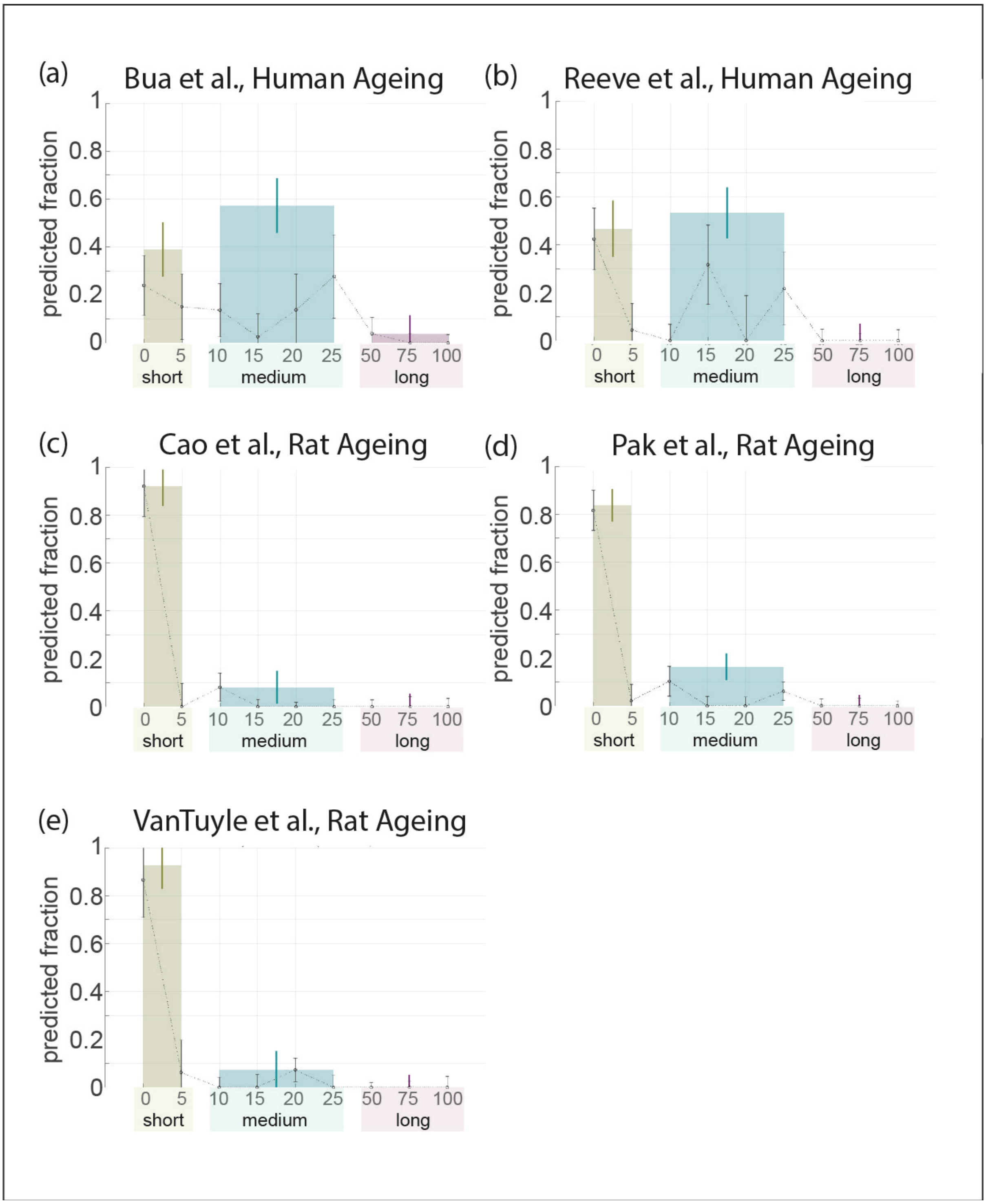

Figure S4. Mixture model analysis using datasets from individual articles.

We performed mixture model analysis on mtDNA deletion datasets reported in individual studies with dataset size above 25 deletions. Example results for individual article datasets from human ageing (a) Bua et al. (Bua et al., 2002), (b) Reeve et al. (Reeve et al., 2008) and rat ageing (c) Cao et al. (Cao et al., 2001), (d) Pak et al. (Pak et al., 2005) and (e) VanTuyle et al. (Van Tuyle et al., 1996) are shown in the figure.

Figure S5. Related to Figures 2 and 3 and Discussion

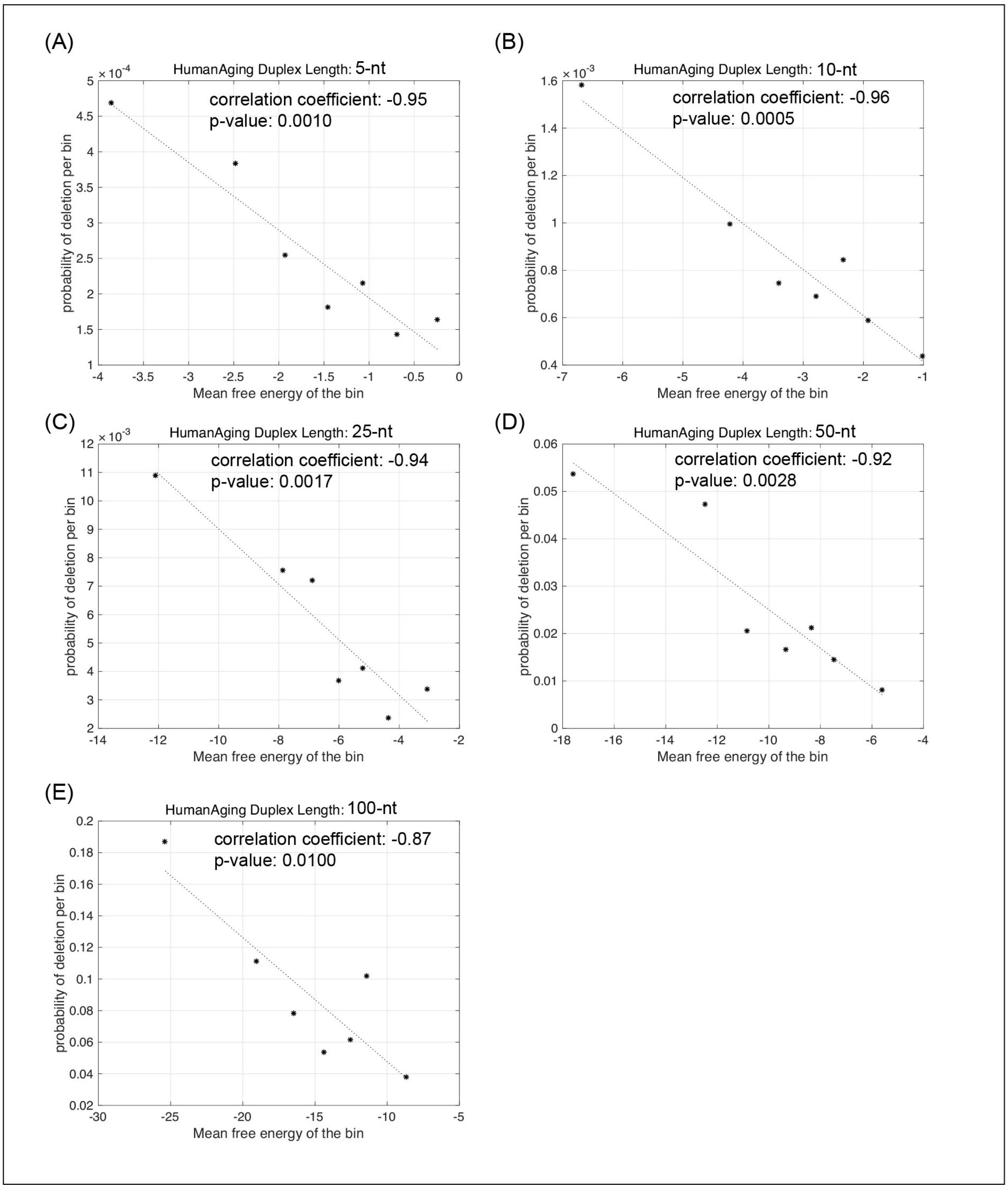

Figure S5. Enrichment analysis of human ageing mtDNA deletion breakpoints.

Guo *et al.* reported higher deletion breakpoint frequencies in regions of human mtDNA that could misalign with higher stability (i.e. more negative Gibbs free energy). The subplots show the frequencies of mtDNA deletion breakpoints in human ageing dataset associated with misalignments of mtDNA of length (A) 5-nt, (B) 10-nt, (C) 25-nt, (D) 50-nt and (E) 100-nt, as a function of the DNA hybridization free energies. Regardless of the segment length, the frequencies of mtDNA deletion breakpoints are higher for mtDNA segments forming more stable hybridization (i.e. more negative Gibbs free energy).

Table S1. Related to Table 1

Dataset count	Dataset Name	Sources
1	Human MTL epilepsy	Volmering et al., 2016
2	Human ageing	Bua et al., 2006, Dimberg et al., 2014, Eshaghian et al., 2006, Fayet et al., 2002, Kraytsberg et al., 2006, Nicholas et al., 2009, Reeve et al., 2008, Zhang et al., 1992
3	Single deletion myopathy	Campbell et al., 2014, Degoul et al., 1991, Ferlin et al., 1997, Grady et al., 2014, Kiyomoto et al., 1997, López-Gallardo et al., 2009, Mita et al., 1990, Ota et al., 1991, Sadikovic et al., 2010, Solano et al., 2003, Wilichowski et al., 1997
4	Human POLG	Wanrooij et al., 2004
5	Inclusion body myositis	Jansson et al., 2000, Moslemi et al., 1997, Rygiel et al., 2016
6	Pearson Syndrome	Ayed et al., 2011, Broomfield et al., 2015, de Vries et al., 1992, Ester Almeida, 2007, Gagne et al., 2014, Knerr et al., 2003, Krauch et al., 2002, Lee et al., 2007, Lohi et al., 2005, Morikawa et al., 1993, Muraki et al., 1997, Park et al., 2015, Rotig et al., 1995, Rotig et al., 1990, Sadikovic et al., 2010, Sato et al., 2015, Shanske et al., 2002, Superti-Furga et al., 1993, Tumino et al., 2011, Williams et al., 2012, Wong, 2001, Yanagihara et al., 2001
7	Charcot Marie Tooth Disease	Vielhaber et al., 2013
8	Rat ageing	Cao et al., 2001, Pak et al., 2005, Van Tuyle et al., 1996, Wanagat et al., 2001
9	Mouse ageing	Chung et al., 1996, Inoue et al., 1997, Inoue et al., 2000, Kim, 1997, Nelson et al., 1993, Vermulst et al., 2008, Wang et al., 1997

10	DSB mouse	Bacman et al., 2009, Fukui and Moraes, 2009, Srivastava and Moraes, 2005
11	WT mouse (NGS)	Pohjoismaki et al., 2013
12	Sod2$^{+/-}$ mouse (NGS)	Pohjoismaki et al., 2013
13	*TWNK*$^{+}$ (NGS)	Pohjoismaki et al., 2013
14	Sod2$^{+/-}$; *TWNK*$^{+}$ (NGS)	Pohjoismaki et al., 2013
15	Monkey ageing	Gokey et al., 2004, Lee et al., 1994, McKiernan et al., 2009
16	*C. elegans* ageing	Lakshmanan et al., 2018

Transparent Methods Section

Deletion breakpoint positions and mitochondrial DNA sequences

We compiled 9,921 breakpoint positions of mtDNA deletions from published literature and grouped them into species- and clinical condition-specific categories (see Supplemental Data). Breakpoints are categorized into distinct groups based on clinical conditions reported in source articles. The complete list of data sources from which the mtDNA breakpoints data were gathered is given in Supplemental Table 1 (Supplemental Information). Deletions reported in normal aged tissues from human, rhesus monkey, mouse, rat and *C. elegans* were grouped under age-related deletions. Disease-associated human mtDNA deletions were further grouped under single deletion myopathy (including Kearns-Sayre syndrome (KSS) and chronic progressive external ophthalmoplegia (CPEO)), myopathy with compound mutations in *polg* gene, Pearson syndrome (PS), Inclusion body myositis, Mesial temporal lobe epilepsy and Charcot Marie tooth disease based on the clinical description of the patients. Our datasets also included mtDNA deletions from mice expressing mitochondrial Pst1/Sca1 restriction enzymes, *Sod2*$^{+/-}$ heterozygous mice, wild type control mice and Twinkle overexpression mice. For mammals, we have considered only the deletions occurring within the mtDNA major arc region, as deletions involving the minor arc were much less frequent. Within each group of mtDNA deletions, identical breakpoint positions were accounted only once. Partially duplicated mtDNA (pd-mtDNA) molecules containing one wild type and one deleted mtDNA portion have been reported to be present together with mtDNA deletions. Rearrangements reported as duplications have been excluded from the datasets in our analysis. Similar to mtDNA deletions, breakpoint junctions of pd-mtDNA molecules have also been reported to have flanking short DR motifs (Bodyak et al., 2001). Hence, pd-mtDNA molecules, if present, could exhibit misalignments signatures similar to that of mtDNA deletions.

In the analyses, we used the complete, annotated mtDNA sequences for human (NC_012920), rhesus monkey (NC_005943), mouse (NC_005089), rat (X14848) and *C. elegans* (NC_001328) from NCBI database. The breakpoint positions were reported based on the L-strand nucleotide sequence.

DNA hybridization partition function calculation

DNA-DNA hybridization partition function (Z) is defined by

$$Z = \sum_i exp\left(\frac{-\Delta G_i^o}{RT}\right)$$

where, ΔG_i^o is the Gibb's free energy of hybridization of the ith conformation (Dimitrov and Zuker, 2004). Multiple conformations could exist between a given pair of hybridizing duplexes and the partition function sums up the probabilities of occurrence of all possible duplex conformations that could form between the given pair of DNA segments. We employed the *hybrid* subroutine of the UNAFold package, setting the temperature to 37° C for mammalian datasets and 25.5° C for *C. elegans* dataset, while keeping the default values for all other parameters (Markham and Zuker, 2008).

Generation of *in silico* breakpoints

In silico dataset is a set of computationally generated breakpoint pair positions with specific hybridization signatures at their breakpoint positions. To generate an *in silico* breakpoint dataset we first set (1) the composition of hybridization lengths θ_l^*, (2) the sample size (i.e., number of deletions), and (3) the window(s) of analysis (i.e. location in mtDNA from where the breakpoints should be drawn). From θ_l^* and the sample size, we determined the number of breakpoint pairs to be sampled from each misalignment length. For each position pair in the window of analysis, we calculated the length specific propensities of

DNA hybridization, as described in the main text. *In silico* breakpoint pair positions were randomly sampled from within the window of analysis using an inverse sampling procedure based on the propensity for each misalignment length *l*-nt in the analysis (Hammersley and Handscomb, 1964). For the validation of our analytical method, we used the window of analysis from human ageing dataset for the generation of *in silico* datasets.

For computing the variance of θ_l^* in the analysis of reported mtDNA deletions, we generated 100 *in silico* datasets using the windows of analysis and the optimal composition θ_l^* in the mtDNA deletion dataset. We analyzed each of the *in silico* datasets to generate 100 *in silico* θ_l^*'s. The variance of θ_l^* was computed as the variance of the 100 *in silico* θ_l^*'s.

Maximum likelihood analysis and statistical analysis

The optimization for likelihood maximization was performed using *fmincon* function in MATLAB (version 2015a; MathWorks, Inc.) with a multi-start strategy using 5 different starting points to ensure that we obtained a globally optimal solution. Statistical analyses were performed using in built functions in MATLAB. Two-sided z-test was used for statistical comparison of log-likelihood values between dataset and *in silico* generated samples. Two-sided *t*-test and Mann Whitney U (MWU) test were used for statistical comparison of mean, median values of breakpoint DR distance values, respectively. Unless stated otherwise, a statistical significance is set at p-value < 0.05.

Implementation of analysis from Guo et al. (2010)

The analysis was performed following the procedure described in the original study by Guo et al (Guo et al., 2010). We performed the analysis for 5, 10, 25, 50 and 100 nt long duplexes. While analyzing short sequence lengths, 35.8% of 820,000 5-nt duplexes and 1.7% of 205,000 10-nt long duplexes had positive values for minimum free energy of hybridization. These

duplexes with positive free energy values were excluded from the analysis. In Guo et al(Guo et al., 2010)., free energy of hybridization assigned to each deletion mutation corresponded to the free energy of the duplex that contained the breakpoint positions. 22.5% (for 5-nt) and 0.6% (for 10-nt) of the 151 human ageing mtDNA deletions fell within duplexes with positive free energy of hybridization and hence were excluded from the analysis. Pearson correlation coefficient and the statistical significance of the linear correlation were calculated using standard subroutines in MATLAB.

Supplemental References

Ayed, I. B., Chamkha, I., Mkaouar-Rebai, E., Kammoun, T., Mezghani, N., Chabchoub, I., Aloulou, H., Hachicha, M. & Fakhfakh, F. 2011. A Tunisian patient with Pearson syndrome harboring the 4.977kb common deletion associated to two novel large-scale mitochondrial deletions. *Biochem Biophys Res Commun,* 411**,** 381-6.

Bacman, S. R., Williams, S. L. & Moraes, C. T. 2009. Intra- and inter-molecular recombination of mitochondrial DNA after in vivo induction of multiple double-strand breaks. *Nucl. Acids Res.,* 37**,** 4218-4226.

Bodyak, N. D., Nekhaeva, E., Wei, J. Y. & Khrapko, K. 2001. Quantification and sequencing of somatic deleted mtDNA in single cells: Evidence for partially duplicated mtDNA in aged human tissues. *Human Molecular Genetics,* 10**,** 17-24.

Broomfield, A., Sweeney, M. G., Woodward, C. E., Fratter, C., Morris, A. M., Leonard, J. V., Abulhoul, L., Grunewald, S., Clayton, P. T., Hanna, M. G., et al. 2015. Paediatric single mitochondrial DNA deletion disorders: an overlapping spectrum of disease. *J Inherit Metab Dis,* 38**,** 445-57.

Bua, E., Johnson, J., Herbst, A., Delong, B., Mckenzie, D., Salamat, S. & Aiken, J. M. 2006. Mitochondrial DNA-deletion mutations accumulate intracellularly to detrimental levels in aged human skeletal muscle fibers. *Am J Hum Genet,* 79**,** 469-80.

Bua, E. A., Mckiernan, S. H., Wanagat, J., Mckenzie, D. & Aiken, J. M. 2002. Mitochondrial abnormalities are more frequent in muscles undergoing sarcopenia. *Journal of Applied Physiology,* 92**,** 2617-2624.

Campbell, G., Krishnan, K. J., Deschauer, M., Taylor, R. W. & Turnbull, D. M. 2014. Dissecting the mechanisms underlying the accumulation of mitochondrial DNA deletions in human skeletal muscle. *Hum Mol Genet,* 23**,** 4612-20.

Cao, Z., Wanagat, J., Mckiernan, S. H. & Aiken, J. M. 2001. Mitochondrial DNA deletion mutations are concomitant with ragged red regions of individual, aged muscle fibers: analysis by laser-capture microdissection. *Nucleic Acids Research,* 29**,** 4502-8.

Chung, S. S., Eimon, P. M., Weindruch, R. & Aiken, J. M. 1996. Analysis of age-associated mitochondrial DNA deletion breakpoint regions from mice suggests a novel model of deletion formation. *AGE,* 19**,** 117-128.

De Vries, D. D., Buzing, C. J., Ruitenbeek, W., Van Der Wouw, M. P., Sperl, W., Sengers, R. C., Trijbels, J. M. & Van Oost, B. A. 1992. Myopathology and a mitochondrial DNA deletion in the Pearson marrow and pancreas syndrome. *Neuromuscul Disord,* 2**,** 185-95.

Degoul, F., Nelson, I., Amselem, S., Romero, N., Obermaier-Kusser, B., Ponsot, G., Marsac, C. & Lestienne, P. 1991. Different mechanisms inferred from sequences of human mitochondrial DNA deletions in ocular myopathies. *Nucleic Acids Res,* 19**,** 493-6.

Dimberg, J., Hong, T. T., Skarstedt, M., Löfgren, S., Zar, N. & Matussek, A. 2014. Novel and differential accumulation of mitochondrial DNA deletions in Swedish and vietnamese patients with colorectal cancer. *Anticancer Res,* 34**,** 147-52.

Dimitrov, R. A. & Zuker, M. 2004. Prediction of Hybridization and Melting for Double-Stranded Nucleic Acids. *Biophysical Journal,* 87**,** 215-226.

Eshaghian, A., Vleugels, R. A., Canter, J. A., Mcdonald, M. A., Stasko, T. & Sligh, J. E. 2006. Mitochondrial DNA deletions serve as biomarkers of aging in the skin, but are typically absent in nonmelanoma skin cancers. *J Invest Dermatol,* 126**,** 336-44.

Ester Almeida, H. L., Helena Almeida, Maria Do Céu Machado, Aguinaldo Cabral, Laura Vilarinho 2007. Síndrome de Pearson. Caso clínico. *Acta Pediatrica Portuguesa,* 38**,** 79-81.

Fayet, G., Jansson, M., Sternberg, D., Moslemi, A. R., Blondy, P., Lombès, A., Fardeau, M. & Oldfors, A. 2002. Ageing muscle: clonal expansions of mitochondrial DNA point mutations and deletions cause focal impairment of mitochondrial function. *Neuromuscul Disord,* 12**,** 484-93.

Ferlin, T., Guironnet, G., Barnoux, M. C., Dumoulin, R., Stepien, G. & Mousson, B. 1997. Detection of mitochondrial DNA deletions by a screening procedure using the polymerase chain reaction. *Mol Cell Biochem,* 174**,** 221-5.

Fukui, H. & Moraes, C. T. 2009. Mechanisms of formation and accumulation of mitochondrial DNA deletions in aging neurons. *Hum Mol Genet,* 18**,** 1028-1036.

Gagne, K. E., Ghazvinian, R., Yuan, D., Zon, R. L., Storm, K., Mazur-Popinska, M., Andolina, L., Bubala, H., Golebiowska, S., Higman, M. A., et al. 2014. Pearson marrow pancreas syndrome in patients suspected to have Diamond-Blackfan anemia. *Blood,* 124**,** 437-40.

Gokey, N. G., Cao, Z., Pak, J. W., Lee, D., Mckiernan, S. H., Mckenzie, D., Weindruch, R. & Aiken, J. M. 2004. Molecular analyses of mtDNA deletion mutations in microdissected skeletal muscle fibers from aged rhesus monkeys. *Aging Cell,* 3**,** 319-26.

Grady, J. P., Campbell, G., Ratnaike, T., Blakely, E. L., Falkous, G., Nesbitt, V., Schaefer, A. M., Mcnally, R. J., Gorman, G. S., Taylor, R. W., et al. 2014. Disease progression in patients with single, large-scale mitochondrial DNA deletions. *Brain,* 137**,** 323-34.

Guo, X., Popadin, K. Y., Markuzon, N., Orlov, Y. L., Kraytsberg, Y., Krishnan, K. J., Zsurka, G., Turnbull, D. M., Kunz, W. S. & Khrapko, K. 2010. Repeats, longevity and the sources of mtDNA deletions: evidence from 'deletional spectra'. *Trends Genet,* 26**,** 340-343.

Hammersley, J. M. & Handscomb, D. C. 1964. *Monte Carlo methods,* London, New York, Methuen; Wiley.

Inoue, K., Ito, S., Takai, D., Soejima, A., Shisa, H., Lepecq, J. B., Segal-Bendirdjian, E., Kagawa, Y. & Hayashi, J. I. 1997. Isolation of mitochondrial DNA-less mouse cell lines and their application for trapping mouse synaptosomal mitochondrial DNA with deletion mutations. *J Biol Chem,* 272**,** 15510-5.

Inoue, K., Nakada, K., Ogura, A., Isobe, K., Goto, Y., Nonaka, I. & Hayashi, J. I. 2000. Generation of mice with mitochondrial dysfunction by introducing mouse mtDNA carrying a deletion into zygotes. *Nat Genet,* 26**,** 176-81.

Jansson, M., Darin, N., Kyllerman, M., Martinsson, T., Wahlström, J. & Oldfors, A. 2000. Multiple mitochondrial DNA deletions in hereditary inclusion body myopathy. *Acta Neuropathol,* 100**,** 23-8.

Kim, J.-S. K., Min-Jung; Kwon, in-Sook; Song, Eun-Sook 1997. Multiple Age-Associated Mitochondrial DNA Deletions in Mouse Brain. *BMB Reports,* 30**,** 33-36.

Kiyomoto, B. H., Tengan, C. H., Moraes, C. T., Oliveira, A. S. & Gabbai, A. A. 1997. Mitochondrial DNA defects in Brazilian patients with chronic progressive external ophthalmoplegia. *J Neurol Sci,* 152**,** 160-5.

Knerr, I., Metzler, M., Niemeyer, C. M., Holter, W., Gerecke, A., Baumann, I., Trollmann, R. & Repp, R. 2003. Hematologic features and clinical course of an infant with Pearson syndrome caused by a novel deletion of mitochondrial DNA. *J Pediatr Hematol Oncol,* 25**,** 948-51.

Krauch, G., Wilichowski, E., Schmidt, K. G. & Mayatepek, E. 2002. Pearson marrow-pancreas syndrome with worsening cardiac function caused by pleiotropic rearrangement of mitochondrial DNA. *Am J Med Genet,* 110**,** 57-61.

Kraytsberg, Y., Kudryavtseva, E., Mckee, A. C., Geula, C., Kowall, N. W. & Khrapko, K. 2006. Mitochondrial DNA deletions are abundant and cause functional impairment in aged human substantia nigra neurons. *Nat Genet,* 38**,** 518-20.

Lakshmanan, L. N., Yee, Z., Ng, L. F., Gunawan, R., Halliwell, B. & Gruber, J. 2018. Clonal expansion of mitochondrial DNA deletions is a private mechanism of aging in long-lived animals. *Aging Cell***,** e12814.

Lee, C. M., Eimon, P., Weindruch, R. & Aiken, J. M. 1994. Direct repeat sequences are not required at the breakpoints of age-associated mitochondrial DNA deletions in rhesus monkeys. *Mech Ageing Dev,* 75**,** 69-79.

Lee, H. F., Lee, H. J., Chi, C. S., Tsai, C. R., Chang, T. K. & Wang, C. J. 2007. The neurological evolution of Pearson syndrome: case report and literature review. *Eur J Paediatr Neurol,* 11**,** 208-14.

Lohi, O., Kuusela, A. L. & Arola, M. 2005. A novel deletion in a Pearson syndrome infant with hypospadias and cleft lip and palate. *J Inherit Metab Dis,* 28**,** 1165-6.

López-Gallardo, E., López-Pérez, M. J., Montoya, J. & Ruiz-Pesini, E. 2009. CPEO and KSS differ in the percentage and location of the mtDNA deletion. *Mitochondrion,* 9**,** 314-7.

Markham, N. R. & Zuker, M. 2008. UNAFold: software for nucleic acid folding and hybridization. *Methods Mol Biol,* 453**,** 3-31.

Mckiernan, S. H., Colman, R., Lopez, M., Mark Beasley, T., Weindruch, R. & Aiken, J. M. 2009. Longitudinal analysis of early stage sarcopenia in aging rhesus monkeys. *Experimental Gerontology,* 44**,** 170-176.

Mita, S., Rizzuto, R., Moraes, C. T., Shanske, S., Arnaudo, E., Fabrizi, G. M., Koga, Y., Dimauro, S. & Schon, E. A. 1990. Recombination via flanking direct repeats is a major cause of large-scale deletions of human mitochondrial DNA. *Nucl. Acids Res.,* 18**,** 561-7.

Morikawa, Y., Matsuura, N., Kakudo, K., Higuchi, R., Koike, M. & Kobayashi, Y. 1993. Pearson's marrow/pancreas syndrome: a histological and genetic study. *Virchows Arch A Pathol Anat Histopathol,* 423**,** 227-31.

Moslemi, A. R., Lindberg, C. & Oldfors, A. 1997. Analysis of multiple mitochondrial DNA deletions in inclusion body myositis. *Hum Mutat,* 10**,** 381-6.

Muraki, K., Goto, Y., Nishino, I., Hayashidani, M., Takeuchi, S., Horai, S., Sakura, N. & Ueda, K. 1997. Severe lactic acidosis and neonatal death in Pearson syndrome. *J Inherit Metab Dis,* 20**,** 43-8.

Nelson, I., Gerasimov, S., Marsac, C., Lestienne, P. & Boursot, P. 1993. Sequence analysis of a deleted mitochondrial DNA molecule in heteroplasmic mice. *Mamm Genome,* 4**,** 680-3.

Nicholas, A., Kraytsberg, Y., Guo, X. & Khrapko, K. 2009. On the timing and the extent of clonal expansion of mtDNA deletions: evidence from single-molecule PCR. *Exp Neurol,* 218**,** 316-9.

Ota, Y., Tanaka, M., Sato, W., Ohno, K., Yamamoto, T., Maehara, M., Negoro, T., Watanabe, K., Awaya, S. & Ozawa, T. 1991. Detection of platelet mitochondrial DNA deletions in Kearns-Sayre syndrome. *Invest Ophthalmol Vis Sci,* 32**,** 2667-75.

Pak, J., Vang, F., Johnson, C., Mckenzie, D. & Aiken, J. 2005. MtDNA point mutations are associated with deletion mutations in aged rat. *Exp Gerontol,* 40**,** 209-218.

Park, J., Ryu, H., Jang, W., Chae, H., Kim, M., Kim, Y., Kim, J., Lee, J. W., Chung, N. G., Cho, B., et al. 2015. Novel 5.712 kb mitochondrial DNA deletion in a patient with Pearson syndrome: a case report. *Mol Med Rep,* 11**,** 3741-5.

Pohjoismaki, J. L. O., Williams, S. L., Boettger, T., Goffart, S., Kim, J., Suomalainen, A., Moraes, C. T. & Braun, T. 2013. Overexpression of Twinkle-helicase protects

cardiomyocytes from genotoxic stress caused by reactive oxygen species. *Proceedings of the National Academy of Sciences,* 110**,** 19408-19413.
Reeve, A. K., Krishnan, K. J., Elson, J. L., Morris, C. M., Bender, A., Lightowlers, R. N. & Turnbull, D. M. 2008. Nature of mitochondrial DNA deletions in substantia nigra neurons. *Am J Hum Genet,* 82**,** 228-35.
Rotig, A., Bourgeron, T., Chretien, D., Rustin, P. & Munnich, A. 1995. Spectrum of mitochondrial DNA rearrangements in the Pearson marrow-pancreas syndrome. *Human Molecular Genetics,* 4**,** 1327-1330.
Rotig, A., Cormier, V., Blanche, S., Bonnefont, J. P., Ledeist, F., Romero, N., Schmitz, J., Rustin, P., Fischer, A., Saudubray, J. M., et al. 1990. Pearson's marrow-pancreas syndrome. A multisystem mitochondrial disorder in infancy. *The Journal of Clinical Investigation,* 86**,** 1601-8.
Rygiel, K. A., Tuppen, H. A., Grady, J. P., Vincent, A., Blakely, E. L., Reeve, A. K., Taylor, R. W., Picard, M., Miller, J. & Turnbull, D. M. 2016. Complex mitochondrial DNA rearrangements in individual cells from patients with sporadic inclusion body myositis. *Nucl. Acids Res.,* 44**,** 5313-29.
Sadikovic, B., Wang, J., El-Hattab, A., Landsverk, M., Douglas, G., Brundage, E. K., Craigen, W. J., Schmitt, E. S. & Wong, L.-J. C. 2010. Sequence homology at the breakpoint and clinical phenotype of mitochondrial DNA deletion syndromes. *PLoS One,* 5**,** e15687.
Sato, T., Muroya, K., Hanakawa, J., Iwano, R., Asakura, Y., Tanaka, Y., Murayama, K., Ohtake, A., Hasegawa, T. & Adachi, M. 2015. Clinical manifestations and enzymatic activities of mitochondrial respiratory chain complexes in Pearson marrow-pancreas syndrome with 3-methylglutaconic aciduria: a case report and literature review. *Eur J Pediatr,* 174**,** 1593-602.
Shanske, S., Tang, Y., Hirano, M., Nishigaki, Y., Tanji, K., Bonilla, E., Sue, C., Krishna, S., Carlo, J. R., Willner, J., et al. 2002. Identical mitochondrial DNA deletion in a woman with ocular myopathy and in her son with pearson syndrome. *Am J Hum Genet,* 71**,** 679-83.
Solano, A., Gámez, J., Carod, F. J., Pineda, M., Playán, A., López-Gallardo, E., Andreu, A. L. & Montoya, J. 2003. Characterisation of repeat and palindrome elements in patients harbouring single deletions of mitochondrial DNA. *J Med Genet,* 40**,** e86.
Srivastava, S. & Moraes, C. T. 2005. Double-strand breaks of mouse muscle mtDNA promote large deletions similar to multiple mtDNA deletions in humans. *Hum Mol Genet,* 14**,** 893-902.
Superti-Furga, A., Schoenle, E., Tuchschmid, P., Caduff, R., Sabato, V., Demattia, D., Gitzelmann, R. & Steinmann, B. 1993. Pearson bone marrow-pancreas syndrome with insulin-dependent diabetes, progressive renal tubulopathy, organic aciduria and elevated fetal haemoglobin caused by deletion and duplication of mitochondrial DNA. *Eur J Pediatr,* 152**,** 44-50.
Tumino, M., Meli, C., Farruggia, P., La Spina, M., Faraci, M., Castana, C., Di Raimondo, V., Alfano, M., Pittalà, A., Lo Nigro, L., et al. 2011. Clinical manifestations and management of four children with Pearson syndrome. *Am J Med Genet A,* 155a**,** 3063-6.
Van Tuyle, G. C., Gudikote, J. P., Hurt, V. R., Miller, B. B. & Moore, C. A. 1996. Multiple, large deletions in rat mitochondrial DNA: evidence for a major hot spot. *Mutat Res,* 349**,** 95-107.
Vermulst, M., Wanagat, J., Kujoth, G. C., Bielas, J. H., Rabinovitch, P. S., Prolla, T. A. & Loeb, L. A. 2008. DNA deletions and clonal mutations drive premature aging in mitochondrial mutator mice. *Nat Genet,* 40**,** 392-4.

Vielhaber, S., Debska-Vielhaber, G., Peeva, V., Schoeler, S., Kudin, A. P., Minin, I., Schreiber, S., Dengler, R., Kollewe, K., Zuschratter, W., et al. 2013. Mitofusin 2 mutations affect mitochondrial function by mitochondrial DNA depletion. *Acta Neuropathol,* 125**,** 245-56.

Volmering, E., Niehusmann, P., Peeva, V., Grote, A., Zsurka, G., Altmuller, J., Nurnberg, P., Becker, A. J., Schoch, S., Elger, C. E., et al. 2016. Neuropathological signs of inflammation correlate with mitochondrial DNA deletions in mesial temporal lobe epilepsy. *Acta Neuropathol,* 132**,** 277-288.

Wanagat, J., Cao, Z., Pathare, P. & Aiken, J. M. 2001. Mitochondrial DNA deletion mutations colocalize with segmental electron transport system abnormalities, muscle fiber atrophy, fiber splitting, and oxidative damage in sarcopenia. *Faseb j,* 15**,** 322-32.

Wang, E., Wong, A. & Cortopassi, G. 1997. The rate of mitochondrial mutagenesis is faster in mice than humans. *Mutat Res,* 377**,** 157-66.

Wanrooij, S., Luoma, P., Van Goethem, G., Van Broeckhoven, C., Suomalainen, A. & Spelbrink, J. N. **2004**. Twinkle and POLG defects enhance age-dependent accumulation of mutations in the control region of mtDNA. *Nucl. Acids Res.,* 32**,** 3053-64.

Wilichowski, E., Grüters, A., Kruse, K., Rating, D., Beetz, R., Korenke, G. C., Ernst, B. P., Christen, H. J. & Hanefeld, F. 1997. Hypoparathyroidism and deafness associated with pleioplasmic large scale rearrangements of the mitochondrial DNA: a clinical and molecular genetic study of four children with Kearns-Sayre syndrome. *Pediatr Res,* 41**,** 193-200.

Williams, T. B., Daniels, M., Puthenveetil, G., Chang, R., Wang, R. Y. & Abdenur, J. E. 2012. Pearson syndrome: unique endocrine manifestations including neonatal diabetes and adrenal insufficiency. *Mol Genet Metab,* 106**,** 104-7.

Wong, L. J. 2001. Recognition of mitochondrial DNA deletion syndrome with non-neuromuscular multisystemic manifestation. *Genet Med,* 3**,** 399-404.

Yanagihara, I., Inui, K., Yanagihara, K., Park, Y. D., Tanaka, J., Ozono, K., Okada, S. & Kurahashi, H. 2001. Fluorescence in situ hybridization analysis of peripheral blood cells in Pearson marrow-pancreas syndrome. *J Pediatr,* 139**,** 452-5.

Zhang, C., Baumer, A., Maxwell, R. J., Linnane, A. W. & Nagley, P. 1992. Multiple mitochondrial DNA deletions in an elderly human individual. *FEBS Lett,* 297**,** 34-8.
